# Comparison of serum ischemia modified albumin levels between preeclamptic and healthy pregnant women

**DOI:** 10.61622/rbgo/2024rbgo97

**Published:** 2025-01-23

**Authors:** Dinç Zuhal, Çakar Erbil, Kumru Pınar, Erel Özcan, Neşelioğlu Salim, Cimsit Nilüfer, Boz Gizem

**Affiliations:** 1 Health Sciences University Zeynep Kamil Women’s and Children’s Diseases Training and Research Hospital, Gynecology and Obstetrics Clinic İstanbul Turkey Health Sciences University Zeynep Kamil Women’s and Children’s Diseases Training and Research Hospital, Gynecology and Obstetrics Clinic, İstanbul, Turkey.; 2 Yıldırım Beyazıt University Ankara Bilkent City Hospital Medical Biochemistry Laboratory Ankara Turkey Yıldırım Beyazıt University, Ankara Bilkent City Hospital, Medical Biochemistry Laboratory, Ankara, Turkey.; 3 Cankiri Cerkes State Hospital Gynecology and Obstetrics Clinic İstanbul Turkey Cankiri Cerkes State Hospital, Gynecology and Obstetrics Clinic, İstanbul, Turkey.

**Keywords:** Preeclampsia, Serum albumin, Pregnant women, Parturition, Myocardial ischemia, Biomarkers

## Abstract

**Objective:**

Our aims to compare level of serum ischemia modified albümin(IMA) between healthy and preeclamptic pregnancies and to evaluate the relationship of IMA with preeclampsia, preeclampsia severity and perinatal outcomes.

**Methods:**

Our study is a prospective case-control study. A total of 134 pregnant women (66 preeclamptic and 68 healthy pregnant) between 18-45 years of age and between 24- 41 gestational weeks participated. Serum IMA levels were measured by the Albumin Cobalt Binding (ACB) test.

**Results:**

The mean IMA values were found to be significantly higher in the preeclampsia group compared to the control group (p<0,001). Patients were divided into 3 groups; severe preeclampsia(n=29), non-severe preeclampsia(n=37) and healthy pregnant(n=68). Statistically significant difference was not found between severe preeclampsia and non-severe preeclampsia (p=0.505). The performance of IMA values in predicting the development of preeclampsia among all participants was evaluated with Receiver Operating Characteristic (ROC) analysis. According to the ROC analysis, the best cut-off value at which the maximum area under the curve (AUC) was obtained was found when IMA>0.98(AUC: 0.690 95% Confidence Interval (CI): 0.600-0.781 p<0.001). When IMA threshold value of >0.98 was taken to predict preeclampsia; the sensitivity, specificity, positive predictive value (PPV) and negative predictive value (NPV) were calculated as 65.15%, 64.71%, 64.18%, and 65.67%, respectively.

**Conclusion:**

IMA level may be a useful new marker in recognizing and predicting preeclampsia. However, despite the power of recognizing the disease, serum IMA levels do not give an idea about the severity of the disease. More comprehensive studies are needed in order to use IMA levels in the diagnosis of preeclampsia.

## Introduction

Hypertensive diseases of pregnancy are one of the leading causes of maternal and fetal deaths worldwide. Preeclampsia develops in 4.6 percent of pregnancies worldwide.^(1)^ In the long term, patients diagnosed with preeclampsia are at risk of developing cardiac and renal pathologies. Early diagnosis and treatment of pregnant women at high risk of developing preeclampsia are therefore one of the most important focus groups in obstetrics.^(2,3)^

Screening pregnant women with an effective diagnostic marker for preeclampsia can reduce unnecessary procedures, major healthcare costs, and redundant hospitalizations of pregnant women with suspected or preeclampsia without severe features. By monitoring the progression of the disease, preterm births can be diminished by planning the optimal delivery time. Various biochemical and hematological markers are being to studied predict and monitor preeclampsia. The use of biomarkers such as placental growth factor (PIGF) and soluble fms-like tyrosine kinase-1(sFlt-1) is currently adopted in clinical practice in many settings, and it seems that they are reliable to rule out the diagnosis in suspected cases.^(4)^The degree of hypertension and proteinuria are poor prognostic markers for maternal and fetal outcomes, better diagnostic and prognostic markers are in need.

There are various mechanisms proposed in the literature on the mechanism underlying preeclampsia. Maternal, paternal, and fetal variables potentially account for the hypertensive disorders of pregnancy.^(5)^Data indicate that unbalanced distribution of proangiogenic and antiangiogenic factors produced by the placenta is important in terms of leading to endothelial dysfunction. Angiogenesis is crucial for successful placental development. It has also been found that oxidative stress, inflammation, mineral deficiency and metabolic disorders cause endothelial dysfunction and play a role in the development of preeclampsia. Impaired trophoblast invasion leads to oxidative stress and inflammation.^(6)^ Although the pathophysiology of preeclampsia has not been clearly elucidated, insufficiency of trophoblastic invasion of the spiral arteries plays a role in the development of the process. Therefore, ischemia modified albumin (IMA), which is one of the serum biomarkers of tissue damage and myocardial ischemia, may be a potential biological marker for preeclampsia.^(7)^

Ischemia-induced modification of human serum albumin (HSA) has been proposed as a serum biomarker of myocardial ischemia. Under physiological conditions, the amino terminal end (N-terminal) of HSA binds metals such as cobalt, copper and nickel. During ischemia, several changes occur at the amino terminal end (N-terminal) of HSA, probably caused by reactive oxidative free radicals, which reduces the binding ability of such metals, especially cobalt. This new and chemically changed molecule is called “ischemia modified albumin”.^(8)^

IMA values may increase in normal pregnancy as well. It is thought that this may be due to the physiological oxidative stress state of pregnancy.^(9)^ In normal pregnancy, a distinct adaptive response is observed, including inflammation, endothelial cell activity and maternal coagulation activation, as well as the production of many pro-oxidants and vasoactive substances by the placenta.^(10)^ On the other hand, maternal IMA levels have been observed to increase in the first trimester in women with clinical findings of defective endovascular trophoblast development, who later develop preeclampsia.^(11)^ In this study, we aimed to evaluate whether maternal serum IMA concentrations increase in preeclamptic pregnant women compared to healthy pregnant controls and whether they are associated with the severity of preeclampsia and perinatal outcomes.

## Methods

A hospital-based prospective study was conducted on 134 participants; consisting of 66 pregnant women diagnosed with preeclampsia and 68 healthy pregnant women with singleton pregnancies. After obtaining ethics committee approval, pregnant women aged 18-45 years and with a gestational age between 24-41 weeks, diagnosed with preeclampsia and admitted to the delivery and perinatology services at the Zeynep Kamil Women’s and Children’s Health Training and Research Hospital of the Health Sciences University between August 2021 and February 2022, were included in the study. In the selection of patients, the maternal age and gestational age at the time of blood sampling were matched between the preeclampsia group and the control group. The control group was selected by matching the gestational age at blood sampling and maternal age with the case group. Healthy pregnant women aged 18-45 years and with a gestational age between 24-41 weeks were included in the control group. Pregnant women with comorbid conditions such as diabetes, intrahepatic cholestasis of pregnancy, multiple pregnancies, chronic hypertension, renal disease, heart disease, peripheral vascular disease, or autoimmune disease were excluded from the study. The case and control group patients were evaluated up to the 10th day postpartum. In the assessments of the control group within the first 10 days postpartum, it was observed that preeclampsia did not develop.

The study was commenced after obtaining approval from the Clinical Research Ethics Committee at Zeynep Kamil Women and Children Diseases Training and Research Hospital, as per the decision dated 23/06/2021 and numbered 2021-139.

For accurate measurement of blood pressure, the information in the guideline jointly published by the European Society of Hypertension(ESH) and the European Society of Cardiology(ESC) in 2013 was taken as basis. The blood pressure of each patient was assessed using a standardized measuring technique, which involved evaluating the brachial artery blood pressure after a minimum of 5 minutes of rest in a seated position, with the cuff positioned at heart level. The measurements were conducted by a proficient healthcare team member using a validated instrument, together with the suitable cuff size.

The diagnosis of preeclampsia was based on the standard diagnostic criteria in the guidelines published by the American College of Obstetricians and Gynecologists (ACOG) in 2020.^(3)^ According to this; in a normotensive pregnant woman, after the 20th gestational week has a measurement of systolic blood pressure(sBP) as 140 mmHg and above and diastolic blood pressure(dBP) as 90 mmHg and above in two measurements at rest at least 4 hours apart, or measurement of systolic blood pressure as 160 mmHg and above or a diastolic blood pressure as 110 mmHg or higher and a repeat measurement within minutes at the same levels, and a new onset of one or more of the following:

- Presence of proteinuria of 300 mg or more in 24-hour urine protein, or spot urine protein/creatinine ratio of 0.3 and above, or if quantitative measurements cannot be made or dipstick urine protein of 2+ and above;
- Platelet count <100,000/microliter;
- Serum creatinine>1.1 mg/dL or doubling of creatinine level in the absence of other renal disease;
- Liver enzymes, aspartate aminotransferase (AST) and alanine aminotransferase (ALT), levels must be at least twice the normal concentrations;
- Pulmonary edema;
- New onset and ongoing headache that cannot be explained by alternative diagnoses and does not respond to normal doses of analgesics. (Response to analgesia does not exclude the possibility of preeclampsi);
- Visual symptoms(e.g., blurred vision, flashing lights or sparks, scotomata).^(3)^
- The study excluded pregnant women who had other health comorbidities such as diabetes, gestational cholestasis, multiple pregnancy, chronic hypertension, renal disease, heart disease, peripheral vascular disease, and autoimmune disease. Control group was formed by selecting healthy pregnant women who matched the gestational weeks and demographic features of the preeclampsia group. The medical history of all pregnant women involved in the trial was obtained and the study protocol was completed for all participants. The individuals had obstetrical evaluation and fetal development was assessed based on ultrasonographic data.

The study involved collecting blood samples from pregnant women diagnosed with preeclampsia and healthy pregnant women throughout the 24th to 41st weeks of pregnancy. Following the disinfection of the antecubital area of each patient’s forearm using alcohol-soaked cotton, a 3cc blood sample was collected into anticoagulant-free tubes with a vacutainer. The blood samples were subjected to centrifugation at a speed of 5,000 revolutions per minute for a duration of 5 minutes, within 1 hour after being collected from the patient. Following centrifugation, the isolated serum was transferred to sealed Eppendorf tubes and documented on behalf of the patients. The serum samples obtained from the patients were preserved at a temperature of -40°C until they were analyzed. The study did not include samples that exhibited hemolysis or a lipemic appearance.

Maternal serum levels of IMA were assessed using the albumin cobalt binding assay. The binding affinity of cobalt to albumin diminishes in cases of ischemia. The serum of the patient is combined with 50 mL of cobalt chloride solution at a concentration of 0.1% and left to incubate for a duration of 10 minutes. During this procedure, cobalt forms a complex with albumin. Following incubation, a solution of 1.5ng/mL dithiothreitol(DTT) is introduced and thoroughly mixed for a duration of 2 minutes. This process enables the formation of a pigmented compound between DTT and cobalt that is not attached to albumin. The resulting colored complex is quantified spectrophotometrically at a wavelength of 470 nm. Results were expressed as ABSU.

The analysis of the data in our study was carried out using the SPSS software, version 20.0 SPSS Inc, Chicago, Ill. For descriptive statistics, quantitative variables are given as mean ± standard deviation (SD), median, minimum and maximum value, and categorical variables are given as frequency and percentage. In comparing independent categorical variables, chi-square and Fisher’s exact probability test were used if the expected value in any of the cells in the 2 by 2 tables was less than 5. In comparing categorical variables and quantitative variables; first of all, the suitability of the data to normal distribution was evaluated using the Kolmogorov–Smirnov and Shapiro-Wilk tests. When the data conforms to normal distribution; independent sample T test was used to compare quantitative variables with categorical variables containing two categories, and one-way analysis of variance (ANOVA) was used to compare quantitative variables with variables containing more than two categories. If a difference is detected as a result of one-way analysis of variance; Using appropriate post-hoc methods, it was determined which category or categories caused the difference. When the data is not normally distributed; The Mann-Whitney U test was used to compare quantitative variables with categorical variables containing two categories, and the Kruskal Wallis H test was used to compare quantitative variables with variables containing more than two categories. When a difference is detected as a result of the Kruskal Wallis H test; the categories were compared in pairs using the Mann-Whitney U test to determine which category or categories caused the difference. Spearman Rho correlation was used for examining the relationship between two quantitative variables where the data were not normally distributed. The Spearman correlation coefficient (r value) is interpreted as follows: r value < 0.20: Very weak, r value between 0.20 and 0.39: Weak, r value between 0.40 and 0.59: Moderate, r value between 0.60 and 0.79: Strong, r value between 0.80 and 1.00: Very strong relationship. The ability of IMA levels to predict preeclampsia among the participants included in the study was investigated by ROC Curve analysis and the threshold value was calculated using the Youden Index Method. Sensitivity, specificity, positive and negative predictive values were calculated separately according to the specific threshold value. Results were considered statistically significant when the p-value was less than 0.05.

## Results

In this study, no statistically significant difference was found between the two groups in terms of age, gravida, and gestational age. However, body mass index (BMI) was found to be statistically significantly higher in the preeclampsia group compared to the control group(p<0.001) (Table 1).

**Table 1:** The demographics of the preeclampsia and control groups

Variables	Preeclampsia Group (n=66) Mean± SD Median (Min-Max)	Control Group (n=68) Mean ± SD Median (Min-Max)	p-value
Age	29.5±5.3 29 (19-43)	27.9±4.4 28 (18-19)	0.063
BMI (kg/m2)	32.3±5.7 32.0 (21.8-47.0)	28.9±4.1 29.0 (20.2-37.4)	<0.001*
Gravida	2.1±1.2 2 (1-6)	2.4±1.4 2 (1-7)	0.108
Parity, n(%)			
Primarity	37(56.1)	22(32.4)	0.006
Multiparity	29(43.9)	46(67.6)	
Gestational week of sample collection	34.8±3.4 35.5 (26.0-41.0)	34.9±3.3 34.5 (28.0-41.0)	0.968
Systolic blood pressure (mmHg)	142.0±11.2	110.5±7.8	<0.001*
(SBP)	140.0 (100-170)	110.0 (100-130)	
Diastolic blood pressure (mmHg)	102.9±9.2	68.0±7.1	<0.001*
(DBP)	90.0 (60-110)	70.0 (50-80)	
Mean arterial blood pressure (mmHg)	107.8±9.1	82.2±6.4	<0.001*
(MAP)	106.7 (73.3-130)	83.3(70.0-93.3)	

Kg – kilogram; cm – centimeter; BMI - body mass index; m2 – metersquared; mmHg - mililiter mercury; *Statistically significant at p < 0.05; *SBP, DBP and MAP at the time of admission

Not only serum IMA levels, but also complete blood count and serum biochemical parameters of patients were also compared. Hematocrit, creatinine, uric acid, spot urine protein/creatinine ratio, AST, ALT, Lactate Dehydrogenase (LDH), International normalized ratio (INR) and IMA values were found to be significantly higher in the preeclampsia group. Serum Na value was found to be significantly lower in the preeclampsia group. While the average serum IMA values in the preeclampsia group was 1.02±0.15 ABSU, it was found to be 0.93±0.1 ABSU in the control group. The mean serum IMA values were found to be statistically significantly higher in the preeclampsia group compared to the control group(p<0.001) (Table 2).

**Table 2:** Comparison of laboratory results of preeclampsia and control groups

Comparasion	Preeclampsia Group (n=66) Mean± SD Median (Min-Max)	Control Group (n=68) Mean ± SD Median (Min-Max)	p-value
Hemoglobin (g/dl)	12.1±1.5 11.9 (8.3-15.3)	11.9±3.1 11.7 (8.5-13.9)	0.093
Hematocrit (%)	36.8±4.1 36.3 (26.1-49.4)	34.8±4.5 34.9 (11.5-41.4)	0.036*
Leukocyte (/mm3)	10976.5±2710.4 10680 (5380-20900)	10831.3±3220.4 10430 (5790-23320)	0.445
Platelet (/mm3)	238197±73836 243500(64000-457000)	233485.3±71958.6 225000(128000-423000)	0.476
Fibrinogen (mg/dl)	503.4±95.6 515.0 (265-763)	504.3±86.5 504.5 (276-708)	0.956
Creatinine (mg/dl)	0.6±0.1 0.5 (0.3-1.1)	0.4±0.1 0.4 (0.3-0.6)	<0.001*
Uric Acid (mg/dl)	5.2±1.4 5.4 (2.7-10.0)	3.5±1.2 3.2 (1.6-7.0)	<0.001*
Spot urine protein/Creatinine	1.0±1.2 0.6 (0.1-6.1)	0.1±0.1 0.1 (0.1-0.2)	<0.001*
24 Hour urine protein (mg)	978.4±1198.9 462 (129-60002)	241.5±55.9 241.5 (202-281)	0.002*
Na (mEq/L)	135.7±2.1 136 (131-140)	136.9±1.8 137 (133-141)	0.002*
AST (U/L)	21.4 ±19.2 16 (10-155)	15.6±5.6 14.0 (6-38)	0.002*
ALT (U/L)	18.5±32.8 11.5 (4-263)	10.3±5.8 8 (1-32)	0.008*
LDH (U/L)	248.3±83.6 223 (147-559)	189.3±30.2 192 (127-268)	<0.001*
INR	1.0±0.1 1.0 (0.9-1.5)	1.01±0.1 1.0 (0.9-1.2)	0.003*
IMA (ABSU)	1.02±0.15 1.02 (0.66-1.44)	0.93±0.10 0.94 (0.66-1.16)	<0.001*

Na – Sodium; AST - aspartate aminotransferase; ALT - alanine aminotransferase; LDH - lactate dehydrogenase; INR - international normalized ratio; IMA - ischemia modified albümin; *Statistically significant at p < 0.05 (Laboratory results at the time of admission)

**Figure 1:**
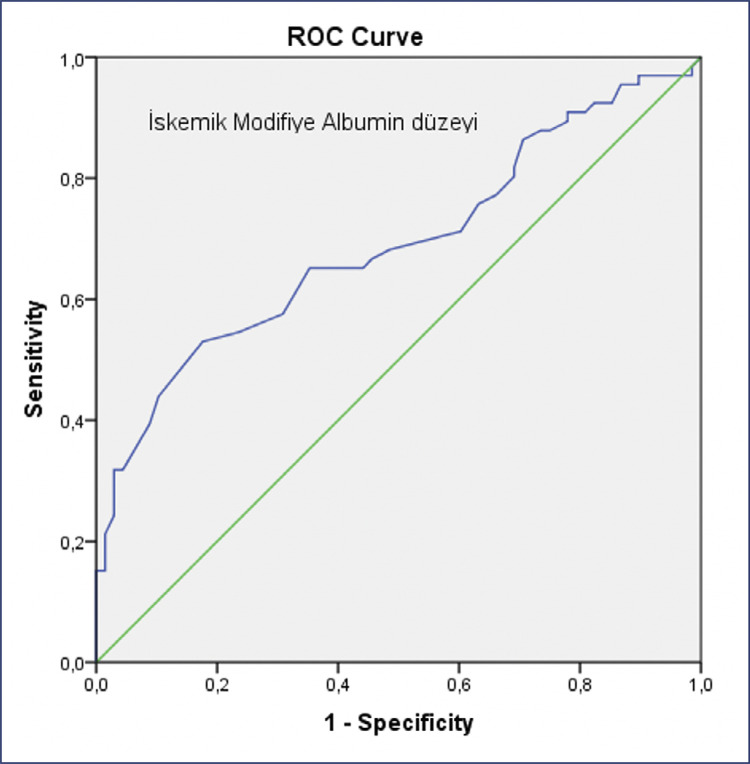
ROC (Receiver Operating Characteristic) curve showing the performance of IMA values in predicting the development of preeclampsia

The performance of IMA values in predicting the development of preeclampsia among all participants included in the study was evaluated by ROC analysis. According to ROC analysis, the best cut-off value at which the maximum area under the curve was determined to be IMA>0.98 (AUC: 0.690 95% CI: 0.600-0.781 p<0.001). When the IMA threshold value was taken as >0.98 in predicting hypertensive diseases; sensitivity, specificity, PPV and NPV were calculated as 65.15%, 64.71%, 64.18% and 65.67%, respectively (Figure 1). When serum IMA level>0.98 ABSU cut-off is taken, preeclampsia is diagnosed 1.9 times more frequently in univariate analysis (OR=1.913(95% CI 0.143-=0.593; p=0.012) (Figure 1).

The preeclampsia group had lower gestational age at birth and birth weight compared to the control group. The need for NICU for the newborn was found to be statistically significantly higher in the preeclampsia group(p<0.001). In babies born to the preeclampsia group, the female gender was found to be significantly higher to the control group(p=0.018) (Table 3).

**Table 3:** Characteristics of newborns in the preeclampsia and control groups

	Preeclampsia Group (n=66) Mean± SD Median (Min-Max)	Control Group (n=68) Mean ± SD Median (Min-Max)	p-value
Gestational week at birth	36.1±3.0 37 (27-41)	38.3±1.9 39 (31-41)	<0.001*
Birth weight (grams)	2456.1±850.5 2565 (720-3750)	3123.9±435.0 3070 (2200-4100)	<0.001*
Preterm birth, n(%)	32(48.5)	6(8.8)	<0.001*
NICU need, n(%)	28(44.4)	8(11.9)	<0.001*
Fetal sex, n(%)			
Female	43(65.2)	30 (44.8)	0.018*
Male	23(34.8)	37 (55.2)	

NICU - Newborn Intensive Care Unit; *Statistically significant at p < 0.05

The relationship between serum IMA levels of pregnant women in the preeclampsia and control groups and continuous variables was assessed using Spearman Rho correlation analysis. A weak positive correlation was detected between serum IMA levels and mean arterial pressure (r=0.324 p<0.001). A weak positive correlation was found between serum IMA levels and platelet count (r= 0.264 p= 0.002). A weak positive relationship was detected between serum IMA and creatinine levels (r= 0.316 p<0.001). Likewise, a positive weak relationship was found between AST (r = 0.228 p = 0.008) and LDH (r = 0.305 p = 0.002) values and serum IMA. A negatively very weak relationship was found between INR and serum IMA (r = -0.197 p = 0.024). A weak negative correlation was found between fetal birth weight and IMA (r=-0.204 p= 0.019) (Table 4).

**Table 4:** The relationship between serum IMA levels in the preeclampsia and control groups and continuous variables

	Serum IMA Level (ABSU)	
	r-value	p-value
Body mass index	0.148	0.087
Gestational week	0.038	0.663
Mean arterial pressure	0.324	<0.001*
Hemoglobin	-0.76	0.380
Leukocyte	0.091	0.293
Platelet	0.264	0.002*
Fibrinogen	0.162	0.077
Creatinine	0.316	<0.001*
AST	0.228	0.008*
ALT	0.188	0.029*
LDH	0.305	0.002*
INR	-0.197	0.024*
Fetal birth weight	-0.204	0.019*

*Statistically significant at p < 0.05

## Discussion

Hypertensive disorders of pregnancy are a prominent contributor to maternal and fetal mortality on a global scale. The global incidence of preeclampsia is 4.6 percent in overall pregnancies.^(1)^ Preeclampsia is a progressive condition that affects multiple bodily systems. It is defined by the sudden development of high blood pressure and the presence of proteinuria during the later stages of pregnancy or early postpartum. It can also present without proteinuria but rather with end organ dysfunction. Consequently, identifying and treating pregnant women with a high likelihood of developing preeclampsia at an early stage is a crucial objective in the field of obstetrics.

IMA is created from the alteration of albumin, induced by reactive oxygen radicals generated as a consequence of ischemia. The albumin cobalt binding test is used to assess serum IMA. This test is also employed for the early identification of ischemia prior to the occurrence of non-transient myocardial injury. While the connection between acute coronary syndrome and IMA has been documented in the literature, the association with preeclampsia is still a topic of discussion and remains uncertain.

The meta-analysis conducted by Seshadri Reddy et al. (2018)^(12)^ examined and compared the levels of serum or fetal cord IMA in pregnant women diagnosed with preeclampsia, healthy pregnant women, and the healthy non-pregnant population. In the analysis, it was shown that the levels of serum or fetal cord IMA were considerably elevated in both the preeclampsia group and the group of individuals with healthy pregnancies, when compared to the non-pregnant population. Simultaneously, this meta-analysis revealed that individuals in the preeclampsia group had notably elevated levels of serum or cord IMA compared to those with normal pregnancies. Assessing serum and cord IMA levels in preeclampsia patients could serve as a valuable, straightforward, cost-effective, and encouraging indicator for evaluating the oxidative stress status in these individuals. It was recommended that further extensive research be carried out to forecast the severity of preeclampsia.^(12)^The study conducted by Üstün et al.^(7)^ involved categorizing pregnant women into three categories: severe preeclampsia (18 patients), mild preeclampsia (18 patients), and healthy pregnant women(18 patients). These groups were then compared based on their serum IMA levels. The serum IMA levels were considerably elevated in pregnant women with preeclampsia compared to those with a healthy pregnancy. Furthermore, there was a positive correlation between the severity of the condition and the serum levels of IMA.^(7)^ In our study; the IMA values of patients in the preeclampsia group (1.02±0.15 ABSU) were found to be significantly higher than those in the control group(0.93±0.10 ABSU)(p<0.001). However, there was no significant correlation between the severity of the disease and the IMA value. This outcome appears consistent with the existing body of literature. The correlation seen between the severity of preeclampsia and serum IMA levels in the study done by Üstün et al.^(7)^ could be attributed to the study’s small sample size. van Rijn et al.^(10)^ examined the serum IMA values of 12 non-pregnant women, 12 normotensive pregnant women, and 12 preeclamptic pregnant women. However, they did not find any significant correlation between these variables.^(10)^ This mismatch is believed to stem from the constraint on the patient population size.

In a 2019 meta-analysis conducted by Seshadri Reddy et al.,^(13)^ the data from six studies were evaluated to determine the sensitivity and specificity of serum IMA value in detecting preeclampsia. The analysis found that the sensitivity of the IMA value was 80% (95% confidence interval: 0.73-0.86), indicating its ability to accurately identify cases of preeclampsia. The specificity was determined to be 76% (95% confidence interval: 0.70-0.81), suggesting its ability to correctly identify non-preeclamptic cases. Additionally, the AUC in the ROC curve was found to be 0.860, indicating that the IMA level may serve as a reliable predictive marker for preeclampsia.^(13)^ In our study, the sensitivity and specificity of serum IMA levels in predicting pregnancy-related preeclamptic diseases were shown to be lower according to the literature. Hence, extensive prospective research is required to investigate this subject in a more complete manner.

In a 2016 study conducted by Bahinipati et al.,^(14)^ serum IMA levels were examined between 40 women with healthy pregnancies and 41 non-pregnant women. The study found that pregnant women had higher serum IMA levels compared to non-pregnant women. In addition, in this study, it was stated that oxidative stress developing from post-ischemic reperfusion is important in normal pregnancy development. The study conducted by Bahinipati et al.^(14)^ highlights that serum IMA, an indicator of cardiac ischemia, increases in healthy pregnant women. Therefore, it is important to continuously monitor serum IMA values during pregnancy to assess the progression of pregnancy. Any deviation or increase in IMA values may indicate complications during pregnancy.^(14)^Further research is required to investigate this topic using a more extensive sample size. When examining literature studies, it is shown that serum IMA levels, which rise in pregnant women compared to non-pregnant women, tend to increase even higher in pregnant women with preeclampsia compared to healthy pregnant women. IMA, a serum marker, can be readily recognized and its regular assessment allows for close monitoring of abnormal rises in serum levels. Furthermore, it serves as an indicator for potential pathological diseases that may arise in the subsequent weeks. Anticipating these issues in advance enables timely intervention and the potential to prevent or minimize the negative impact on health and the likelihood of death. One of the limitations in our study is the absence of a non-pregnant patient group among the participants, and the fact that only one measurement of serum IMA level was observed in all the pregnant women included in the study. In future studies on this matter, non-pregnant groups can also be included in the research, and instead of observing only a single serum IMA level, the regular monitoring of serum IMA levels and the changes between these values can also be the focus of research. Another limitation of our study is the lack of evaluation of serum albumin levels. Further studies could explore the correlation between the Serum IMA/Albumin ratio and preeclampsia.^(15,16)^

We examined various hematological and biochemical parameters, as well as serum IMA levels, in the pregnant women who took part in our study. The study conducted by Makuyana et al.^(17)^ discovered no significant disparity in albumin, bilirubin, and ALT levels between pregnant women diagnosed with preeclampsia and healthy pregnant women. However, preeclampsia was associated with elevated levels of AST and ALP. Among renal function tests levated levels of urea, uric acid, and creatinine were observed in preeclampsia patients when compared to healthy pregnant individuals. No significant disparity in hemoglobin, hematocrit levels, and platelet count was noted between the two groups.^(17)^ In the study conducted by Delić et al.,^(18)^ a comparison was made between complete blood count and biochemistry parameters in 34 pregnant women diagnosed with preeclampsia and 35 healthy pregnant women. The results showed that pregnant women diagnosed with preeclampsia had significantly higher levels of BUN and creatinine, while their platelet count was lower compared to healthy pregnant women. Nevertheless, there were no discernible discrepancies observed in the AST/ALT, LDH, GGT, total bilirubin, hemoglobin, hematocrit, and leukocyte levels.^(18)^ Upon evaluating laboratory measures between the preeclampsia and control groups in our study, no statistically significant disparity was observed in hemoglobin, leukocyte, platelet, and fibrinogen values. Our investigation revealed that the preeclampsia group had significantly elevated levels of hematocrit, creatinine, uric acid, spot urine protein/creatinine ratio, 24-hour urine protein levels, AST/ALT, LDH, and INR compared to the control group. Furthermore, the preeclampsia group exhibited a considerably lower serum sodium levels in comparison to the control group.

## Conclusion

According to our data, the serum IMA levels of pregnant women diagnosed with preeclampsia showed a significant increase compared to the healthy pregnant group. However, it has been found that serum IMA levels are not associated with the severity of preeclampsia. Our study indicates that a single serum IMA measurement is not sufficient to diagnose preeclampsia. More comprehensive studies are needed in order to use IMA levels in the diagnosis of preeclampsia. Also, as serum IMA appears to be inversely weak relationship to birth weight, it could be interesting to conduct a study evaluating whether there is a relationship between serum IMA and neonatal outcomes. Tracking serum IMA values can be especially helpful in preventing complications that may develop due to oxidative damage. This allows earlier prediction of complications, earlier intervention, and prevention or reduction of morbidity and mortality. Looking at similar changes in serum IMA levels in similar studies in the literature, we can conclude that the IMA partially represents disorders in placental perfusion.
